# Use of the Ponseti method for recurrent clubfoot following posteromedial release

**DOI:** 10.4103/0019-5413.38584

**Published:** 2008

**Authors:** Sumeet Garg, Matthew B Dobbs

**Affiliations:** St Louis Children's Hospital, Shriners Hospital St Louis, USA

**Keywords:** Recurrent clubfoot, posteromedial release, Ponseti method, tibialis anterior, tendon transfer

## Abstract

**Background:**

A child with recurrent or incompletely corrected clubfoot after previous extensive soft tissue release is treated frequently with revision surgery. This leads to further scarring, pain and limitations in range of motion. We have utilized the Ponseti method of manipulation and casting and when indicated, tibialis anterior tendon transfer, instead of revision surgery for these cases.

**Materials and Methods:**

A retrospective review of all children treated since 2002 (*n* = 11) at our institution for recurrent or incompletely corrected clubfoot after previous extensive soft tissue release was done. Clinical and operative records were reviewed to determine procedure performed. Ponseti manipulation and casting were done until the clubfoot deformity was passively corrected. Based on the residual equinus and dynamic deformity, heel cord lengthening or tenotomy and tibialis anterior transfer were then done. Clinical outcomes regarding pain, function and activity were reviewed.

**Results:**

Eleven children (17 feet) with ages ranging from 1.1 to 8.4 years were treated with this protocol. All were correctable with the Ponseti method with one to eight casts. Casts were applied until the only deformities remaining were either or both hindfoot equinus and dynamic supination. Nine feet required a heel cord procedure for equinus and 15 required tibialis anterior transfer for dynamic supination. Seven children have follow-up greater than one year (average 27.1 months) and have had excellent results. Two patients had persistent hindfoot valgus which required hemiepiphyseodesis of the distal medial tibia.

**Conclusion:**

The Ponseti method, followed by tibialis anterior transfer and/or heel cord procedure when indicated, can be successfully used to correct recurrent clubfoot deformity in children treated with previous extensive soft tissue release. Early follow-up has shown correction without revision surgery. This treatment protocol prevents complications of stiffness, pain and difficulty in ambulating associated with multiple soft tissue releases for clubfeet.

## INTRODUCTION

The Ponseti method has been repeatedly shown to provide excellent correction of infantile clubfoot deformity. The results of Ponseti have been reproduced successfully by a number of surgeons at varied locations of the world.^1^–^12^ In most places it is the preferred method for treatment of clubfoot deformity. Surgical treatment, however, still is used frequently when relapses occur. All reported series of clubfeet treated with the Ponseti method describe substantial recurrence rates of up to 41%.^1^–^12^

Ponseti utilized the tibialis anterior tendon transfer to the lateral cuneiform to treat early recurrence. This treatment converts the muscle from an inversion force to an eversion force on the mid and forefoot and corrects dynamic supination of the foot during gait. The goal is to correct deformity before it becomes fixed. Several reports have shown excellent results with long-term maintenance of correction of the relapsed clubfoot after tibialis anterior transfer.^13^–^16^

A more difficult problem to treat than the recurrent non-operated clubfoot is the persistently deformed clubfoot after extensive soft tissue release. Treating recurrent clubfoot after surgery puts the physician in a quandary. It is known that initial surgery leads to poor outcomes with significant pain, stiffness and fatigue. Repeated surgery has the potential to worsen these symptoms by further postoperative scar formation. As initially described by Garceau and modified by Ponseti, tibialis anterior transfer was designed as a treatment for dynamic deformity, not fixed deformity.^9^,^16^ Use of this procedure in a fixed deformity provides suboptimal results.

Nevertheless, spurred by the excellent results of tibialis anterior transfer for dynamic deformity in children we attempted to utilize this procedure in the failed surgical clubfoot by converting the fixed deformity into a dynamic deformity utilizing Ponseti's principles. Older children presenting to our clinic with a recurrent clubfoot after extensive soft tissue releases were treated with manipulation and casting until correction of the fixed deformity (with exception of hindfoot equinus). They then underwent tibialis anterior transfer to the lateral cuneiform and if necessary, heel cord lengthening or tenotomy. In this series we describe the technique and early results of this treatment protocol.

## MATERIALS AND METHODS

**Figure 1:**
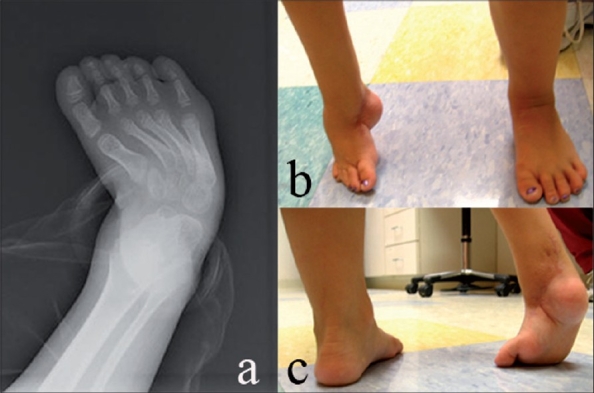
A five-year-old child with recurrent right clubfoot, depicting preoperative deformity. (a) AP X-ray of right foot with ankle showing forefoot adduction. (b,c) Clinical appearance of right foot showing forefoot supination adduction and hind foot equinus

Institutional review board approval was obtained for a retrospective review of clinical and operative records for patients treated for clubfoot recurrence after previous surgical release. Records were reviewed for cases since 2002. Children were included in the cohort if they presented with a recurrent, fixed clubfoot deformity following posteromedial soft tissue release done elsewhere [Figure 1]. Prior surgical treatment was determined based on parent report and observation of typical surgical scars on affected feet. Many patients had multiple prior surgeries after their posteromedial release. Clinical determination of a recurrent clubfoot was made by the senior investigator. Children were excluded if their deformity was not fixed, in which case they proceeded immediately to tibialis anterior transfer without preoperative manipulation and casting.

Details of surgical treatment were verified by reviewing operative notes. Intraoperative and perioperative complications were noted if present. Clinical results were reviewed to determine both static and dynamic foot position after surgical treatment. Difficulties with shoe wear and ambulation were noted. Integrity of the tendon transfer was assessed on physical examination. Patients were interviewed regarding pain, function and activity as part of routine post-surgical follow-up.

Manipulation and casting was done utilizing the Ponseti method.^17^ Details of the Ponseti method are well described, as is our specific protocol.^5^ Long leg casts were applied and changed weekly until correction of all aspects of the deformity, except equinus and dynamic supination, was complete. Preoperative standing radiographs were taken to evaluate bony anatomy and ability for the midfoot to accept the tendon transfer. Patients were then taken to the operating room to undergo heel cord lengthening (or tenotomy if under the age of four) if they had equinus deformity after casting. Tibialis anterior transfer was then done in all children with dynamic deformity indicated by foot supination during gait. Presence of dynamic supination was based on clinical observation of the senior investigator (MBD). If both heel cord tenotomy/lengthening and tibialis anterior transfer were necessary they were done under a single anesthesia.

We used a modification of Garceau's procedure, as described by Ponseti. The tibialis anterior is sharply incised off its insertion on the medial and plantar aspect of the medial cuneiform. It is then secured with a no. 1 PDS suture weaved through the cut tendon edge in a modified Bunnell fashion. An incision is made dorsally over the lateral cuneiform and dissection is taken down sharply to the bony surface. Extensor tendons are retracted and a ¼ inch drill bit is used to open a channel through the depth of the cuneiform. The tendon is then passed subcutaneously to the lateral incision and taken through the bone channel plantarwards with two free Keith needles. The suture is then tied down over a tendon button on the plantar surface of the foot. The transfer is tensioned in a position of maximal foot abduction and a long leg non-weight bearing fiberglass cast is applied after skin closure. The tendon button is cut at four weeks and the cast is changed to a walking short leg cast with the foot in abduction. After two additional weeks the walking cast is removed and no further bracing is given. Children were able to begin gradual resumption of activities and sports following cast removal.

## RESULTS

**Figure 2:**
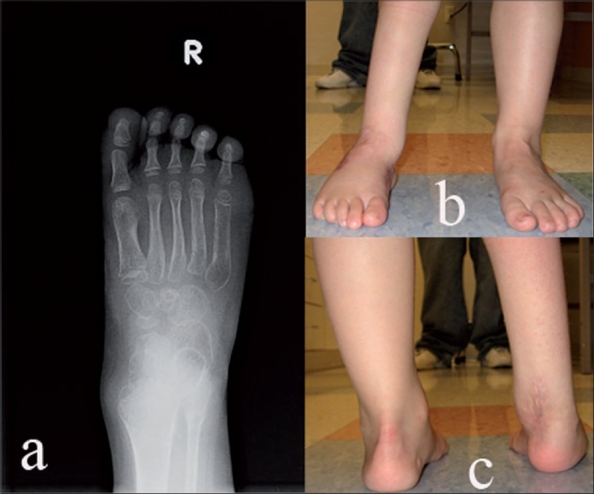
Clinicoradiological deformity correction of the same patient after treatment with manipulation, Achilles tendon lengthening and tibialis anterior transfer. (a) Postoperative AP X-ray of foot with ankle depicting correction of forefoot adduction. (b,c) Clinical appearance of right foot after treatment

Eleven children, six girls and five boys, with ages ranging from 1.1 years to 8.4 years (average 4.6 years) have been treated according to the described protocol in the past five years [Table 1]. They had 17 involved feet. All 11 previously had extensive posteromedial release done on their clubfeet at outside institutions (six had multiple prior surgical procedures) and had presented to our center for treatment of persistent or recurrent deformity. An average of three casts (range one to eight) was needed to achieve full correction of the clubfoot deformity (except for hindfoot equinus). Seven feet required heel cord lengthening and three feet underwent heel cord tenotomy. Tenotomy is utilized instead of lengthening in children under the age of four. Seven feet did not have equinus deformity noted after casting. After manipulation, 15 of 17 feet had dynamic supination with gait and hence underwent tibialis anterior transfer [Figure 2]. Two feet (in one child) were corrected with manipulation and tenotomy alone and did not require tendon transfer. No child had acute complications from the manipulation, casting or surgical procedures.

**Table 1:** Clinical details of patients

ID	Gender	No. of feet	Age (yrs)	No. of previous surgeries	Clinical presentation	No. of cases for correction	Heel cord surgery	Tibialis anterior transfer	Follow-up (months)	Outcome
1	F	2	8.4	2	E C A V S	4	Lengthening	Yes	7.5	Good
2	M	1	2.0	1	E C A V S	5	Tenotomy	Yes	8.0	Good
3	M	2	4.8	1	E S	2	Lengthening	Yes	16.4	Good
4	F	2	1.1	1	E V S	3	Tenotomy	No	65.1	Good
5	F	2	5.5	2	E C A V S	3	Lengthening	Yes	12.4	Good
6	M	1	5.3	2	A S	2	None	Yes	13.1	Persistent hindfoot valgus
7	M	2	5.0	1	A S	2	None	Yes	36.9	Good
8	F	2	4.3	1	A S	3	None	Yes	4.4	Good
9	F	1	4.0	4	E C A V S	6	Lengthening	Yes	8.2	Good
10	F	1	4.6	2	AS	1	None	Yes	25.8	Persistent hindfoot valgus treated with hemiepiphyseodesis
11	M	1	5.5	2	AS	1	None	Yes	20.2	Good

E - Equinus C - Cavus A - Forefoot adductus V - Hindfoot valgus S - Dynamic supination

Results in four children with less than one year of follow-up are preliminary. They all have had excellent initial outcomes with improvements in appearance and gait with no complaints of foot pain. They can all wear standard shoes comfortably. None of these patients had a complication and none have required any further treatment beyond that described. The remainder have greater than one year follow-up (average 27.1 months) and have maintained correction without further manipulation [Figure 1]. Two of these seven had persistent hindfoot valgus deformity; one child underwent a distal medial tibia hemiepiphyseodesis procedure using staples and the other is contemplating proceeding with hemiepiphyseodesis. All have markedly improved their appearance and gait and can wear regular shoes comfortably. None have complaints of foot pain either at rest or with activity.

## DISCUSSION

Many studies have shown poor long-term function after surgical treatment of clubfoot.^18^–^23^ They are frequently stiff, painful and have limited range of motion. Recurrences are usually treated with further surgery, however, this only worsens long-term functional outcomes. Dobbs described a cohort of 45 patients with long-term follow-up after surgical treatment of clubfoot.^21^ Number of surgical procedures was associated with significantly worse functional and radiographic outcomes in patients with clubfeet. Although it is tempting to surgically revise the failed clubfoot after posteromedial release, this evidence suggests that surgery only leads to further functional compromise.

Prior literature has described successful use of the Ponseti method in older infants.^2^,^24^ A recent series from Iowa reported on the use of the Ponseti method in neglected clubfeet in children who were ambulatory. The average age in their series was 3.9 years and 16 of 24 feet were able to be corrected without soft tissue release.^24^ Our center has also had excellent results utilizing the Ponseti method in neglected clubfeet which are often seen in our population of foreign adopted children. Our success in using the Ponseti method in the older child with rigid, neglected clubfoot encouraged us to use the method in children with recurrent clubfeet after soft-tissue release. This was combined with tibialis anterior transfer to correct dynamic supination and when necessary, heel cord procedures to treat equinus. All three of these maneuvers (manipulation and casting, tendon transfer and heel cord lengthening/tenotomy) are part of Ponseti's original description of his method for treating clubfoot.^9^

Garceau first described transfer of the tibialis anterior for recurrent clubfoot deformity in 1940.^25^ In his report the tendon was transferred laterally to either the base of the fifth metatarsal or the cuboid. He described generally good results, however, had several feet overcorrected into valgus. Ponseti modified Garceau's procedure by transferring the tibialis anterior tendon into the lateral cuneiform as opposed to the lateral border of the foot. This was felt to correct the dynamic nature of the recurrence with reduced risk of overcorrection into valgus. Tibialis anterior transfer is an important part of the Ponseti method for treatment of clubfoot. In his initially reported cohort, 53% of his patients underwent tibialis anterior transfer, with no detrimental outcomes in long term.^9^ The high rate of recurrence in his initial cohort was due to lack of emphasis on post-correction bracing, now a well-known risk factor for recurrence.

A recent study by Farsetti *et al.*, in 2006 described long-term results following tibialis anterior transfer for recurrent clubfeet.^14^ In their series, the tendon transfer was effective in treating dynamic deformity, however, was not effective in treating static deformity. Feet that were not passively correctable prior to tibialis anterior transfer had significantly worse outcomes than those whose feet were passively correctable. Our treatment protocol for the recurrent clubfoot after surgical soft tissue release incorporates the finding from Farsetti's work. Our goal prior to performing the tibialis anterior transfer is to passively correct the deformity such that what remains is a dynamic deformity. The equinus deformity is readily corrected if necessary with a simple heel cord lengthening and does not violate any foot or ankle articulations. The short-term results demonstrated in this series support this treatment protocol. By avoiding repeat surgical realignment of the clubfoot the long-term complications of repeated clubfoot surgery are avoided.

While it may at first appear daunting to apply the Ponseti method in stiff, recurrent clubfeet, the principles remain the same regardless of age. Manipulation is actually easier to perform since the anatomy of the foot is much easier to appreciate in the larger child. We have been able to use the Ponseti method successfully at our institution in older children, even those with recurrent deformity or prior surgery. The Ponseti method, incorporating manipulation, casting and when needed, heel cord tenotomy/lengthening and tibialis anterior transfer can successfully treat the recurrent clubfoot after previous operative treatment. The major drawback of this treatment, as in Garceau's series, remains overcorrection of the hindfoot deformity into valgus by pull of the transferred tendon. We have seen this complication in two patients in this early series and have thus far successfully treated one with hemiepiphyseodesis utilizing staples. The other patient is considering the same procedure.

When faced with the dilemma of a child with recurrent clubfoot after prior extensive soft tissue release one should not immediately repeat soft-tissue releases. The path of continued surgery has several pitfalls and poor long-term outcomes. We acknowledge that our data is preliminary with short follow-up, but thus far it has proven to be a well accepted and tolerated treatment for recurrent clubfoot. Although there are a few families who adamantly desire repeat surgery, most are very eager to pursue our more conservative treatment plan. We plan to continue annual follow-up on our treated children through skeletal maturity and are continuing to utilize this treatment protocol at our institution.
